# Potential Role of Vitamin C in the Associations of Serum Per- and Polyfluoroalkyl Substances with Bilirubin and Albumin in U.S. Adolescents and Young Adults: NHANES 2011–2018

**DOI:** 10.3390/antiox15101310

**Published:** 2026-10-12

**Authors:** Chan Li, Kexin Zhu, Zhongzheng Niu, Heather Ochs-Balcom, Diana S. Aga, Lisa Lenker, Marina Feiler, Changxing Ma, Mindula K. Wijayahena, Lina Mu

**Affiliations:** 1 Department of Epidemiology and Environmental Health, School of Public Health and Health Professions, University at Buffalo, The State University of New York, Buffalo, NY 14214, USA; chanli@buffalo.edu (C.L.); kexinzhu@buffalo.edu (K.Z.); zniu2@buffalo.edu (Z.N.); hmochs2@buffalo.edu (H.O.-B.); marinaok@buffalo.edu (M.F.); 2 Department of Chemistry, College of Arts and Sciences, University at Buffalo, The State University of New York, Buffalo, NY 14260, USA; dianaaga@buffalo.edu (D.S.A.); mindulaw@buffalo.edu (M.K.W.); 3 Research and Education in Energy, Environment and Water (RENEW) Institute, University at Buffalo, The State University of New York, Buffalo, NY 14260, USA; lisalenk@buffalo.edu; 4 Department of Biostatistics, School of Public Health and Health Professions, University at Buffalo, The State University of New York, Buffalo, NY 14214, USA; cxma@buffalo.edu

**Keywords:** PFAS, bilirubin, albumin, vitamin C, NHANES, adolescents, survey analysis, BKMR

## Abstract

Research on per- and polyfluoroalkyl substances (PFAS) and biomarkers potentially related to oxidative stress in adolescents is limited. We investigated associations between serum PFAS and total bilirubin and albumin among 1474 participants aged 12–20 years using National Health and Nutrition Examination Survey 2011–2018 data. Six primary PFAS and two secondary measures, summed branched perfluorooctanesulfonic acid (Sm-PFOS) and total PFOS, were assessed. Survey-weighted linear regression with multiple imputation for missing covariates was used to assess associations and potential vitamin C effect modification. Bayesian kernel machine regression was used to evaluate PFAS mixture associations. After adjustment for multiple comparisons, perfluorooctanoic acid (PFOA), perfluorohexane sulfonic acid (PFHxS), and perfluorodecanoic acid (PFDeA) were positively associated with bilirubin, and seven PFAS measures were positively associated with albumin. Albumin associations for PFOA, perfluorononanoic acid (PFNA), and PFDeA were stronger among vitamin C-deficient participants (<11.4 µmol/L), with significant interactions. For example, the PFOA–albumin association was stronger among vitamin C-deficient than non-deficient participants (β = 2.276 vs. 0.544; interaction *q* = 0.013). Mixture associations were positive for both biomarkers and consistent with the primary analysis. These findings suggest PFAS are associated with bilirubin and albumin; vitamin C findings are exploratory and limited by the small deficient group.

## 1. Introduction

Per- and polyfluoroalkyl substances (PFASs) are a large group of synthetic chemicals that have been extensively incorporated into industrial applications and consumer products since the mid-20th century [1]. They are highly persistent and ubiquitous in the environment. Children and adolescents may encounter PFASs through a variety of exposure routes, including contaminated food and drinking water, household materials, personal care products, prenatal and early-life exposures, and inhalation of PFAS-related precursor compounds [2]. Biomonitoring data indicate that PFASs remain widely detectable in the U.S. population, and exposure has been associated with multiple health outcomes, including endocrine and liver-related effects [1,3,4]. These exposures are especially concerning during childhood and adolescence, which are periods of rapid growth and physiological development. In adolescents, PFAS exposure has been associated with hormone variation and reduced serum folate concentrations [3,5].

PFASs are chemically complex. Many commonly measured PFASs can exist as both linear and branched structural isomers [6]. PFOS is one example: in addition to linear PFOS, several branched PFOS isomers have been identified. These individual branched compounds are structural isomers of PFOS, whereas Sm-PFOS refers to their summed concentration rather than a single isomer [6,7]. Distinguishing linear from branched PFOS isomers may be important because prior studies suggest differences in environmental distribution, bioaccumulation, toxicokinetics, serum half-lives, and elimination [6,7,8]. Isomer measurement and analysis may therefore provide additional insight into exposure history and internal dose patterns that are not fully captured by a single PFOS measure. However, whether branched PFOS isomers differ in their associations with biomarkers potentially related to oxidative stress remains poorly understood.

Experimental evidence suggests that PFASs can perturb redox regulation, the balance between reactive species and antioxidant defenses [9,10]. Reviews by Bonato et al. [10] and Fenton et al. [11] summarize experimental and human evidence while highlighting differences among compounds, species, and exposure conditions. The systematic review and meta-analysis by Costello et al. integrates rodent experiments and human observational evidence on PFAS-related liver injury, an endpoint distinct from the biomarkers examined here [12]. For example, Suh et al. observed reactive oxygen species generation, cardiolipin oxidation, and mitochondrial dysfunction in PFOA-treated RIN-m5F cells, a rat pancreatic β-cell-derived cell line [13]. These findings provide biological plausibility under experimental conditions. Bilirubin and albumin participate in antioxidant defense, but their serum concentrations also reflect physiological processes beyond redox regulation and are not direct measures of oxidative damage [14,15,16,17,18,19,20,21,22].

Adolescence is a critical developmental period during which redox regulation may have particular relevance to metabolic and immune health [23]. Although associations of PFASs with bilirubin, albumin, oxidative/nitrative markers, and related inflammatory outcomes have been studied in adult and other populations [24,25,26,27,28], epidemiological evidence in adolescents remains limited. PFASs continue to be detected despite declining concentrations of several legacy compounds, underscoring the ongoing ubiquity and complexity of exposure [4,29].

Because several PFAS are highly persistent, bioaccumulative in the human body, and resistant to degradation, reducing the internal body burden after exposure is difficult [4,8]. Therefore, identifying factors that may influence PFAS-related biological responses is important. Vitamin C (L-ascorbic acid) is an essential micronutrient that donates electrons and participates in antioxidant recycling, as discussed in reviews of its chemistry and physiology [30,31,32,33,34]. Human studies of air pollution, including a trial of combined vitamin C and E supplementation and an observational study of vitamin C intake, provide indirect evidence relevant to antioxidant status [35,36]. PFOA-exposed mouse studies also provide a rationale for examining vitamin C [37,38]. However, these studies do not establish a protective effect against PFAS in humans, and evidence remains limited on whether serum vitamin C modifies PFAS–biomarker associations in adolescents.

We examined associations between serum PFAS concentrations and two biomarkers potentially related to oxidative stress, total bilirubin and albumin, among U.S. adolescents and young adults aged 12–20 years, and evaluated whether these associations differed by serum vitamin C status. We also examined Sm-PFOS and total PFOS to characterize associations involving branched PFOS isomers. This study addresses the limited epidemiological evidence on PFASs and biomarkers potentially related to oxidative stress during adolescence and early adulthood, including the potential modifying role of vitamin C status.

## 2. Methods

### 2.1. Study Population

NHANES is a multistage probability survey of the U.S. civilian noninstitutionalized population [39]. We used data from four cycles: 2011–2012, 2013–2014, 2015–2016, and 2017–2018. Among 5601 participants aged 12–20 years, 1479 had complete PFAS measurements. The lower age limit corresponded to PFAS laboratory eligibility in NHANES 2011–2012 [40]. The age range includes adolescence, defined by the World Health Organization as 10–19 years, and early adulthood [41]. Five participants with both bilirubin and albumin missing were excluded, leaving a final sample of 1474 participants with both bilirubin and albumin measurements. Participants with missing covariates were included through multiple imputation. Figure S1 shows participant selection and the subsets used for secondary PFOS and vitamin C analyses.

### 2.2. Biomarker Measurements

Total bilirubin (mg/dL) and albumin (g/L) were measured in the serum collected at NHANES mobile examination centers. These biomarkers were selected because of their roles in antioxidant defense: bilirubin scavenges reactive species, while albumin contributes through its binding properties and redox-active thiol groups [14,15]. Laboratory procedures and quality-control protocols are documented by NHANES [39]. Outcome-specific eligibility required an available measurement of the biomarker being analyzed.

### 2.3. PFAS Measurement

Serum PFAS concentrations were quantified using high-performance liquid chromatography–tandem mass spectrometry [40,42]. The six primary serum exposures were perfluorooctanesulfonic acid (PFOS), perfluorooctanoic acid (PFOA), perfluorohexane sulfonic acid (PFHxS), perfluorononanoic acid (PFNA), 2-(N-methyl-perfluorooctane sulfonamido) acetic acid (Me-PFOSA-AcOH), and perfluorodecanoic acid (PFDeA). The PFOS and PFOA measures represented total concentrations in 2011–2012 and linear n-PFOS and n-PFOA in later cycles; the 2013–2014 linear measurements came from the surplus-isomer file [40,42]. Secondary analyses evaluated Sm-PFOS and total PFOS in 2015–2018 (*n* = 715). Sm-PFOS represents the summed branched PFOS isomers, and total PFOS was calculated as linear PFOS plus Sm-PFOS. We used the NHANES-released concentrations, including values below the limit of detection (LOD) replaced by LOD/√2, and the corresponding detection indicators.

### 2.4. Serum Vitamin C Measurement

Serum vitamin C was measured using high-performance liquid chromatography with electrochemical detection [43]. Measurements were available in NHANES 2017–2018, expressed in µmol/L [43]. Of the 334 eligible participants examined in that cycle, 332 had measured vitamin C. The other 1140 participants were examined in cycles without vitamin C measurements and did not contribute to the vitamin C analyses.

Serum vitamin C was categorized as deficient (<11.4 µmol/L) or non-deficient (≥11.4 µmol/L) using the biochemical deficiency threshold described by the Centers for Disease Control and Prevention (CDC) [44]. This threshold identifies deficiency rather than adequate vitamin C status. The vitamin C analyses were exploratory and were not prospectively prespecified. Analyses were restricted to participants with measured vitamin C; missing values were not imputed.

### 2.5. Covariates

Covariates were selected based on the previous literature, causal considerations, and the survey design. The adjustment set included continuous age, sex, race/ethnicity, household-reference education, family income, serum cotinine category, total cholesterol, and survey cycle. Demographic and socioeconomic characteristics were considered potential common determinants of PFAS exposure and biomarker concentrations; cotinine represented tobacco-smoke exposure. Total cholesterol was included to account for lipid status and related metabolic and hepatic processes that may influence serum PFAS, bilirubin, and albumin concentrations. Because PFAS may also affect cholesterol, its causal role is uncertain, and the adjusted coefficients are interpreted as associations conditional on cholesterol and the other covariates. Body mass index (BMI) was summarized descriptively; its uncertain causal position could make adjustment susceptible to overadjustment. Race/ethnicity, education, family income, cotinine, and cycle were categorical. Refused or unknown education and income responses were treated as missing.

Missing covariates were handled using multiple imputation by chained equations with 50 imputations and 20 iterations [45,46]. Education and family income were imputed using augmented multinomial models; cholesterol was imputed using predictive mean-matching with five donors. Partially reported income ≥$20,000 was restricted to compatible categories. Predictors included outcomes, the laboratory PFAS panel, age and age squared, sex, race/ethnicity, cotinine, family and household size, log sampling weight, and survey primary sampling unit (PSU) indicators representing strata and cycle [47]. Exposure-specific vitamin C imputation models also included measured vitamin C and exposure/outcome-by-vitamin-C terms. Only missing covariate values were imputed. This approach assumes missingness at random conditional on the imputation predictors.

### 2.6. Statistical Analysis

Descriptive analyses summarized participant characteristics by survey cycle and PFAS concentrations across participant characteristics. Survey-weighted analyses incorporated masked strata (SDMVSTRA), primary sampling units (SDMVPSU), and PFAS subsample weights [48,49]. Primary analyses used WTSA2YR in 2011–2012, WTSSBH2Y in 2013–2014, and WTSB2YR in 2015–2018, divided by four to combine the cycles. WTSSBH2Y accounted for the surplus-sample eligibility required for the 2013–2014 PFOS/PFOA measurements [42]. Secondary 2015–2018 analyses used WTSB2YR divided by two; vitamin C analyses used unscaled 2017–2018 WTSB2YR. Counts and vitamin C group percentages are unweighted unless otherwise stated.

Survey-weighted linear regression estimated crude and adjusted associations of each PFAS with bilirubin and albumin. PFAS concentrations were natural log-transformed, and β coefficients represented the absolute change in each biomarker per one-unit increase in ln(PFAS), equivalent to an approximately 2.7-fold increase in concentration. PFAS concentrations were also grouped into low, medium, and high categories based on tertiles of their distributions, using mean ranks for ties; category ranges and counts are shown in Table S1. The low category was the reference. Primary models included 1474 participants, and secondary Sm-PFOS and total PFOS models included 715 participants. Adjusted models included the covariates described above. Taylor-linearized variances accounted for strata, and estimates were combined using Rubin’s rules with finite-sample degrees of freedom.

Vitamin C was examined as a potential effect modifier using models containing ln(PFAS), vitamin C-deficiency group, their interaction, and covariate main effects. Group-specific slopes were estimated from these models with common covariate coefficients. Wald tests assessed the difference between the non-deficient and deficient slopes. Benjamini–Hochberg false discovery rate (FDR) correction [50] was applied separately to 12 primary tests (six PFASs × two biomarkers) and four secondary tests within each continuous, category-trend, and interaction analysis; *q* < 0.05 indicated statistical significance.

Spearman correlations described relationships among PFASs (Table S4). Bayesian kernel machine regression (BKMR) evaluated the joint association of the six primary PFASs with each biomarker while allowing nonlinear exposure–response relationships and interactions among mixture components [51]. Log-transformed PFAS and outcomes were standardized, and models included the same adjustment variables, a full Gaussian kernel, and component-wise variable selection. Each outcome model included 1474 participants and used 10,000 iterations, 5000 burn-in iterations, and thinning by 10. The reporting fit used the first completed imputation, and a second fit used the second completed imputation to assess between-fit variation. Sm-PFOS and total PFOS were not included in the mixture model. Posterior summaries were specific to each fit and were not pooled across 50 imputations. Mixture contrasts compared joint exposure quantiles with the 50th percentile. Posterior inclusion probabilities (PIPs) represented the relative inclusion of each PFAS conditional on the other mixture components. Effective sample sizes and variation in PIPs were examined (Table S5). BKMR was unweighted and characterized associations within the analytic sample.

## 3. Results

The analysis included 1474 participants aged 12–20 years, with 715 contributing to secondary Sm-PFOS and total PFOS analyses (Figure S1). Participant characteristics and concentrations by survey cycle are shown in Table S2 and Table S3. PFAS concentrations were generally higher among males and varied by age, race/ethnicity, and family income (Table 1). Education, family income, and total cholesterol were missing for 61 (4.1%), 117 (7.9%), and two (0.1%) participants, respectively.

Table 2 presents the associations between natural log-transformed PFAS concentrations and each biomarker. After covariate adjustment, PFOA, PFHxS, and PFDeA were positively associated with total bilirubin, with β coefficients of 0.057 (95% CI: 0.026, 0.089), 0.026 (0.006, 0.047), and 0.047 (0.014, 0.081), respectively. PFOS, PFOA, PFHxS, PFNA, and PFDeA were positively associated with albumin, as were Sm-PFOS (β = 0.635; 95% CI: 0.207, 1.062) and total PFOS (β = 0.597; 95% CI: 0.177, 1.018). These ten associations were significant after adjustment for multiple comparisons. Me-PFOSA-AcOH was not clearly associated with either biomarker, and the bilirubin associations for PFOS, PFNA, Sm-PFOS, and total PFOS were not significant. Using LOD/2 in place of LOD/√2 for concentrations below detection yielded estimates consistent with the primary analysis, with the same significant overall associations (Table S6).

**Figure 1:**
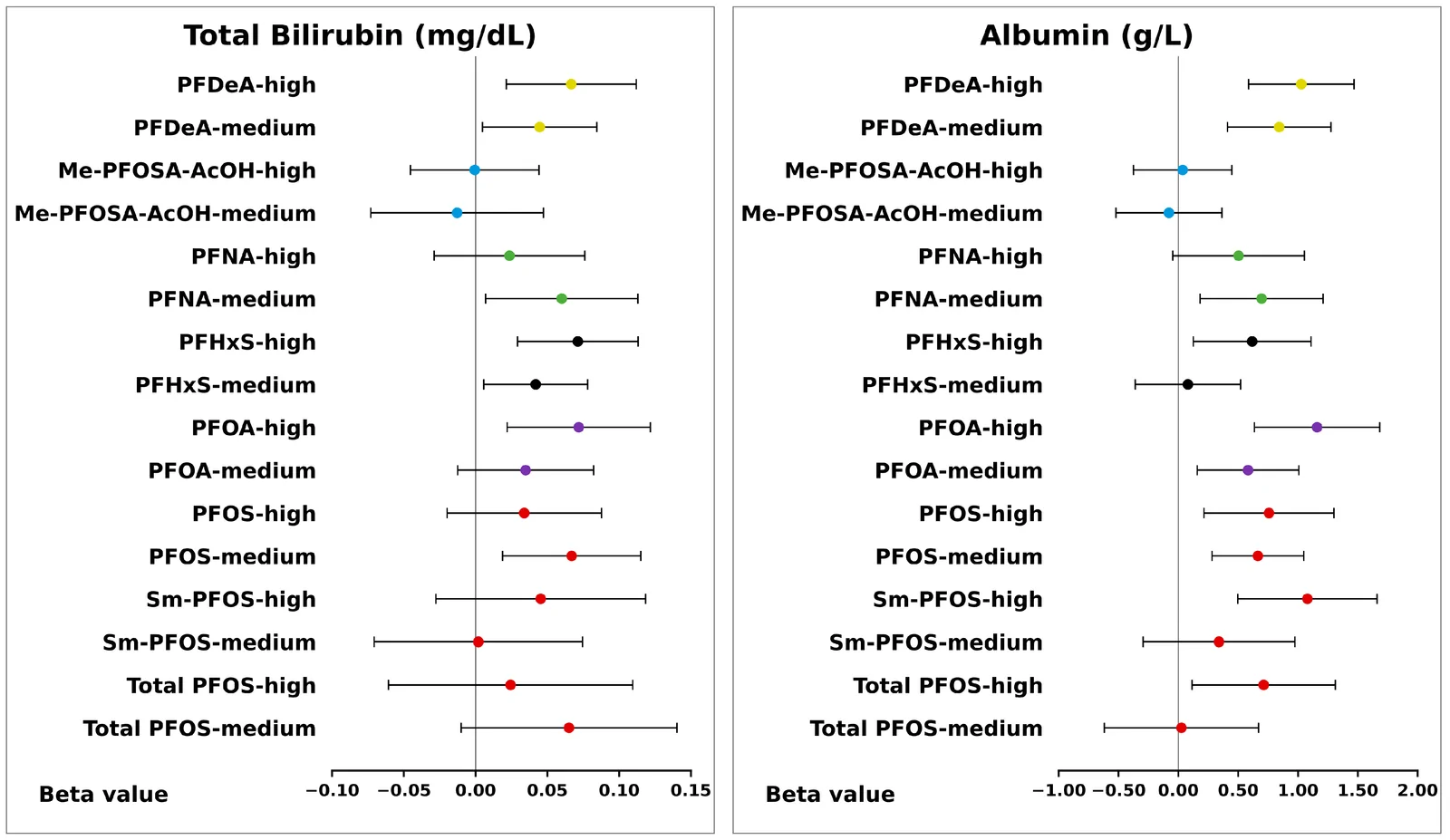
Survey-weighted associations of categorical PFAS concentrations with total bilirubin and albumin. Each panel shows adjusted β estimates for medium- and high-PFAS categories relative to the low category; black error bars represent 95% confidence intervals. Colors identify PFAS measures: red, PFOS, Sm-PFOS, and total PFOS; purple, PFOA; black, PFHxS; green, PFNA; blue, Me-PFOSA-AcOH; yellow, PFDeA. Models adjusted for age, sex, race/ethnicity, household-reference education, family income, survey cycle, cholesterol, and cotinine. Primary models used *n* = 1474; Sm-PFOS and total PFOS models used *n* = 715 (2015–2018). Missing covariates were multiply imputed. Table S1 gives exact category ranges and counts.

Figure 1 shows the adjusted associations of medium- and high-PFAS categories with each biomarker, relative to the low category. For total PFOS, the albumin estimate was larger in the high category, while the medium-versus-low contrast was close to zero (β = 0.026; 95% CI: −0.619, 0.671; *p* = 0.935).

**Figure 2:**
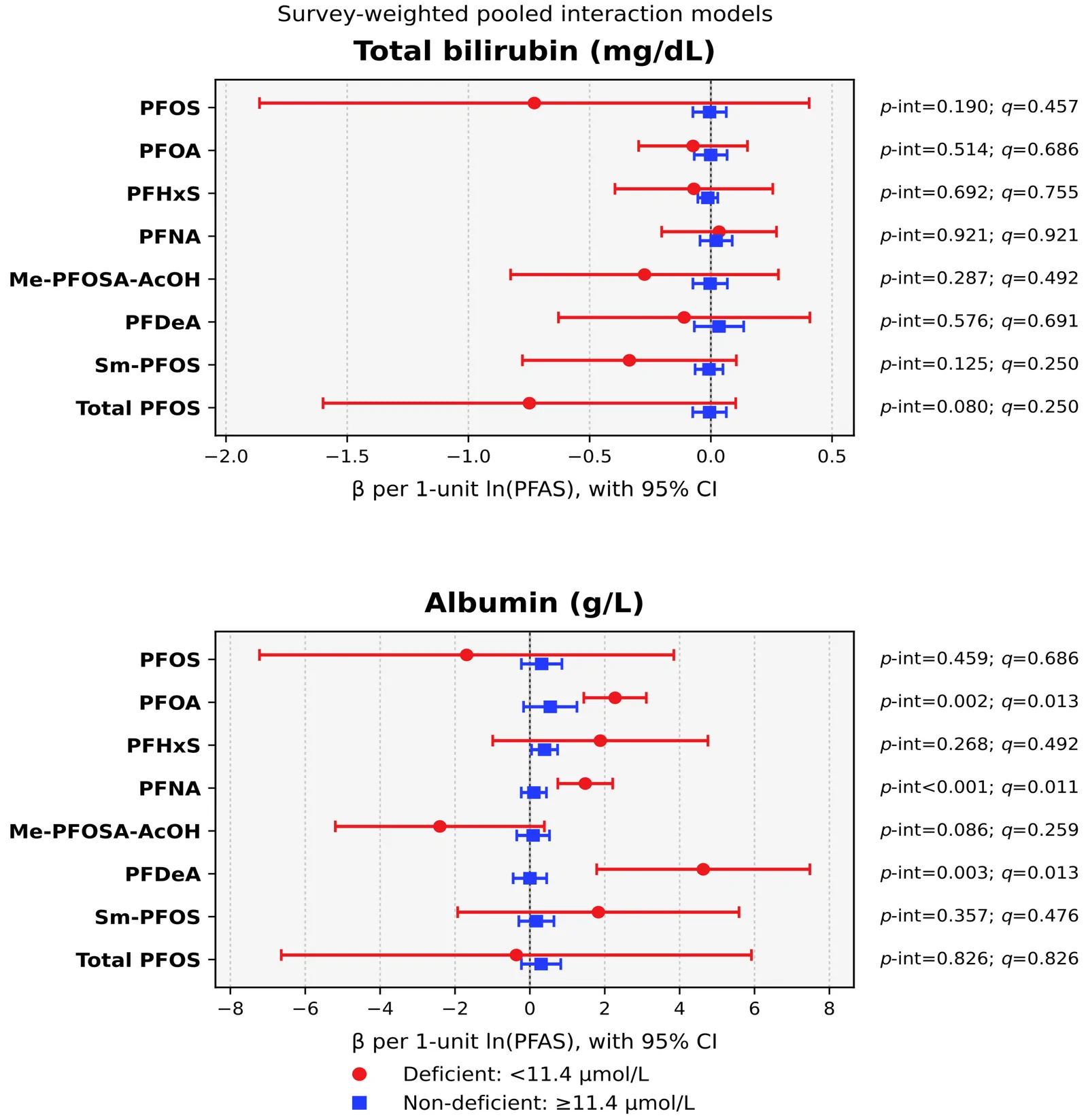
Survey-weighted associations of eight PFAS measures by vitamin C status: deficient (<11.4 µmol/L) and non-deficient (≥11.4 µmol/L). Models included 332 participants with measured vitamin C. Points and bars show adjusted group-specific slopes and 95% confidence intervals. *p*-int denotes the interaction *p*-value; *q* denotes the Benjamini–Hochberg-adjusted *p*-value.

Among the 332 participants with measured vitamin C, less than 10% had concentrations <11.4 µmol/L. The albumin associations for PFOA, PFNA, and PFDeA were stronger in the deficient group (Table 2; Figure 2). Deficient versus non-deficient β coefficients were 2.276 versus 0.544 g/L for PFOA, 1.479 versus 0.106 g/L for PFNA, and 4.631 versus 0.001 g/L for PFDeA per unit ln(PFAS). The corresponding interaction *p*-values were 0.002, <0.001, and 0.003, with *q*-values of 0.013, 0.011, and 0.013. No bilirubin interaction was significant.

**Figure 3:**
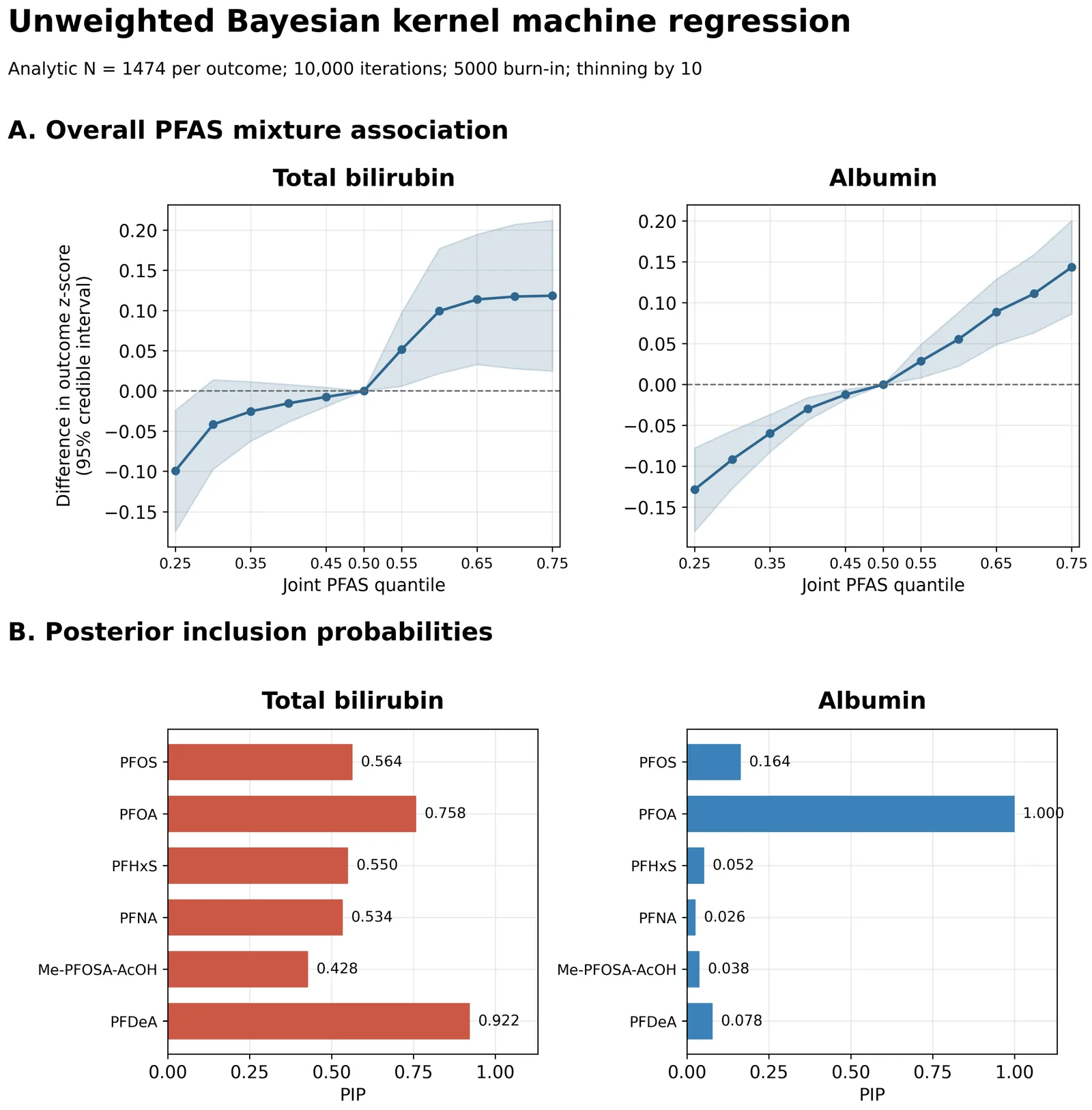
Unweighted BKMR results (*n* = 1474). Blue lines and points in panel A show posterior mean changes in standardized bilirubin and albumin when all six ln-transformed PFASs are moved together to each quantile relative to their medians; light blue shaded bands show approximate 95% credible intervals. The dashed horizontal line indicates zero difference. They are not compound-specific exposure–response curves. Numerical PIPs indicate conditional inclusion, not causal effects. Results were not survey-weighted or pooled across imputations.

Figure 3 shows positive joint-mixture contrasts at the 75th versus 50th percentile. The estimated contrast was 0.118 (approximate 95% credible interval: 0.025, 0.212) standardized units for bilirubin and 0.144 (0.087, 0.201) for albumin. The highest PIPs were PFDeA (0.922), PFOA (0.758), and PFOS (0.564) for bilirubin, and PFOA (1.000), PFOS (0.164), and PFDeA (0.078) for albumin (Table S5).

## 4. Discussion

Our study examined associations between PFAS and two biomarkers potentially related to oxidative stress among U.S. adolescents and young adults aged 12–20 years. PFOA, PFHxS, and PFDeA were positively associated with total bilirubin, while PFOS, PFOA, PFHxS, PFNA, PFDeA, Sm-PFOS, and total PFOS were positively associated with albumin after FDR correction. Category-based analyses supported several of these associations, and the PFAS mixture showed positive contrasts for both biomarkers. Exploratory analyses suggested differences by vitamin C status: PFOA, PFNA, and PFDeA had stronger positive albumin slopes in the deficient group. Interpretation of these exploratory findings is limited by the small number of vitamin C-deficient participants.

In our 2011–2018 adolescent and young-adult sample, PFAS concentrations were generally higher among males and varied by race/ethnicity, age, and income. This pattern is consistent with NHANES biomonitoring studies showing that serum PFAS concentrations vary by sex, age, and race/ethnicity, and that legacy PFAS levels, particularly PFOS and PFOA, have declined over time after major production changes [3,29,52]. Compared with earlier NHANES cycles, the lower concentrations of several legacy PFASs in younger populations likely reflect national temporal declines rather than the absence of exposure; persistent detection still suggests ongoing exposure, as well as persistence of earlier-life exposure for PFASs with long biological half-lives [2,8,29]. Adults have generally shown higher concentrations of several PFASs than adolescents in NHANES trend analyses, consistent with cumulative exposure, although age differences may also reflect birth-cohort effects and changing exposure sources over time [29]. The higher concentrations observed among males may reflect differences in exposure patterns, diet, product use, and sex-related elimination pathways [2,7,8]. The observed racial/ethnic differences should not be interpreted as intrinsic biological differences, but may reflect variation in exposure opportunities, diet, residential or environmental context, and consumer product use. Comparisons across survey cycles must also consider the use of total PFOS/PFOA in 2011–2012 and linear-isomer measurements in later cycles; the secondary total PFOS analysis was restricted to 2015–2018.

Epidemiological evidence using bilirubin and albumin remains limited in younger populations, so the closest comparisons come from adult or general-population studies. For PFOA, our positive associations with both biomarkers were broadly consistent with those of Omoike et al., who reported positive associations with total bilirubin and albumin among U.S. adults, and with those of Gleason et al., who reported positive associations with total bilirubin among NHANES participants [24,25]. PFOS was positively associated with albumin in our study, whereas its bilirubin association was not significant. Borghese et al. did not observe consistent associations with total bilirubin in Canadian adults, although PFOS was associated with total bilirubin after excluding participants with reported liver disease; albumin was not evaluated [26]. Our PFHxS findings also agreed with Omoike et al., whereas PFNA was associated with albumin but not clearly with bilirubin [24]. For PFDeA, comparisons were more limited: Omoike et al. reported an association with albumin, Gleason et al. did not evaluate PFDeA, and Borghese et al. did not evaluate PFDeA with total bilirubin or examine albumin [24,25,26]. Overall, PFOA showed the most consistent pattern across these studies and our findings. Differences in populations, exposure distributions, and model specifications may contribute to the variation observed for other PFASs. Our study extends this evidence to adolescents and young adults.

Experimental studies of PFASs report changes in reactive oxygen species, mitochondrial function, and antioxidant responses, but translation to humans requires attention to compound-specific toxicokinetics and exposure conditions [10,11,13]. Bilirubin radical scavenging was demonstrated in cell-free biochemical experiments [14], and reviews by Creeden et al. [17] and Vitek et al. [18] describe its broader metabolic and signaling roles. Reviews by Roche et al. and Turell et al. explain albumin’s ligand-binding, radical-scavenging, and Cys34-dependent redox functions [20,21]. Lepedda et al. examined Cys34 oxidative modification in human plasma and carotid-plaque material [15]; this is distinct from measuring total serum albumin. These biomarkers also have broader relevance to health: bilirubin has been studied in relation to pediatric metabolic conditions [19], and a hospital-based human study linked albumin concentrations with inflammatory indices [16]. Bilirubin concentrations reflect heme metabolism and hepatic handling [17], whereas albumin concentrations also depend on hepatic synthesis, nutritional and inflammatory conditions, hydration, and renal losses [16,53]. The review by Soeters et al. also describes how inflammation alters albumin distribution and turnover [22]. In vitro studies using human serum albumin demonstrate PFAS binding [54,55], providing another possible explanation for the albumin findings because protein binding may influence circulating PFAS concentrations and distribution [11]. The observational evidence on urinary oxidative/nitrative DNA markers in Taiwanese adults [27] concerns a different biological endpoint. Thus, the present bilirubin and albumin measurements cannot establish oxidative injury or a compensatory antioxidant response.

Vitamin C status is biologically relevant to antioxidant defense, but the evidence supporting its role requires distinction among experimental systems. Reviews by Njus et al. and Traber and Stevens describe electron donation, radical scavenging, and recycling within antioxidant networks [30,32]. Padayatty and Levine emphasize that vitamin C concentrations depend on intake, absorption, tissue transport, and renal handling, and that experimental effects must be interpreted in relation to physiological concentrations [33]. The review by Carr and Maggini describes vitamin C’s antioxidant and immune-cell functions [34]. The review of PFAS and vitamin metabolism further describes possible interactions between exposure and vitamin-related pathways [31]. In PFOA-exposed mice, Li et al. found that vitamin C attenuated liver enlargement and liver-enzyme elevations and increased hepatic glutathione [37]. Nie et al. reported attenuation of PFOA-related changes in spleen index and white blood cells in another mouse study [38]. These animal experiments support biological plausibility; they do not establish that naturally occurring serum vitamin C differences alter PFAS toxicity in humans or that supplementation would benefit the study population.

Consistent with this mechanistic evidence, human studies of other oxidant environmental exposures suggest that vitamin C or antioxidant status may modify pollution-related biological responses. A randomized, double-blind trial found that combined vitamin C and E supplementation attenuated ozone-related reductions in small-airway function and peak expiratory flow among children with asthma in Mexico City, particularly those with moderate or severe asthma [35]. A cross-sectional study in Korean adults found that the association between higher air-pollution scores and prehypertension or hypertension was stronger among those with lower vitamin C intake [36]. These studies provide indirect support for the hypothesis, although the trial did not isolate vitamin C from vitamin E and the dietary study did not establish causality.

In our analysis, PFOA, PFNA, and PFDeA had stronger positive associations with albumin among participants with vitamin C deficiency. This pattern is compatible with differences in antioxidant availability, but it was not consistent across PFASs or biomarkers. No bilirubin interaction was significant. Less than 10% of participants with measured vitamin C were deficient, limiting the precision of these exploratory estimates. Biological and measurement differences may also help explain the findings. The 11.4-µmol/L cutoff identifies biochemical deficiency [44], but it has not been established as a threshold for PFAS-related effects in adolescents. Vitamin C intake has a nonlinear relationship with plasma and cellular concentrations [56], which a binary classification may not fully capture. A single serum measurement may also incompletely represent the antioxidant status relevant to PFASs that persist for years [8]. Furthermore, experimental studies assessed defined vitamin C interventions and liver or immune injury, whereas our study evaluated naturally occurring serum concentrations and routine biomarkers. These differences limit direct comparison with experimental findings and support further investigation using repeated vitamin C measurements and direct measures of oxidative damage.

The different patterns across compounds and biomarkers argue against interpreting the results as a uniform PFAS-related redox response. For Sm-PFOS and total PFOS, differences between the albumin and bilirubin findings may reflect their distinct physiological determinants and PFAS–protein binding, rather than a common measure of oxidative injury [17,53,54,55]. The limited findings for Me-PFOSA-AcOH also warrant attention because frequent values below detection restrict the exposure information available for this compound. Sm-PFOS combines several branched isomers, so opposing or unequal contributions from individual isomers may be obscured [6,7]. A summed measure cannot establish the behavior of every branched isomer or identify a specific exposure route. Linear and branched PFOS isomers differ in environmental behavior, bioaccumulation, excretion, and serum half-lives [6,7,8]. For example, after cessation of a contaminated drinking-water exposure, Li et al. reported mean half-lives of 2.73 years for linear PFOS, 2.67 years for 2/6m-PFOS, 3.43 years for 3/4/5m-PFOS, and 5.01 years for 1m-PFOS [8]. The smaller secondary sample and aggregation of branched isomers also limit what can be inferred from the weaker findings. These results do not establish that particular compounds are biologically inactive. Larger studies with individual-isomer measurements and direct oxidative-damage biomarkers are needed to distinguish exposure and toxicokinetic differences from biological responses.

This study has several strengths, including its focus on adolescents and young adults, evaluation of individual PFASs and mixtures, and assessment of measured vitamin C and summed branched PFOS isomers. Several limitations should also be noted. The cross-sectional design precludes establishing temporal or causal relationships between PFAS exposure, vitamin C status, and bilirubin or albumin concentrations; residual confounding remains possible. Bilirubin and albumin are influenced by physiological processes beyond oxidative stress, and single measurements may not reflect long-term status. Selection into the PFAS laboratory sample and outcome availability may introduce bias; multiple imputation additionally depends on the missing-at-random assumption. Differences between total and linear PFOS/PFOA measurements across cycles complicate pooled comparisons, and secondary isomer analyses were limited to 2015–2018. Values below detection constrain exposure contrasts for some compounds. The vitamin C analyses were exploratory and limited by the small sample size, with deficiency present in less than 10% of participants with measured vitamin C. High PFAS observations may influence regression estimates. Finally, BKMR was unweighted. Low bilirubin kernel-scale effective sample sizes and variation in PIPs limit confidence in the mixture findings; a single chain per dataset also limits assessment of convergence.

## 5. Conclusions

Several serum PFASs were positively associated with total bilirubin and albumin among U.S. adolescents and young adults. PFOA, PFNA, and PFDeA showed stronger positive albumin associations in the vitamin C-deficient group. These exploratory findings suggest possible variation in PFAS–biomarker associations by vitamin C status, with uncertainty related to the small deficient group and cross-sectional design. Longitudinal and mechanistic studies incorporating repeated vitamin C measurements and direct redox biomarkers are needed to clarify these relationships.

## Figures and Tables

**Table 1:** Weighted PFAS concentration (ng/mL) by participant characteristics.

Parameter	Weighted Mean (SE), ng/mL							
	PFOS	PFOA	PFHxS	PFNA	Me-PFOSA-AcOH	PFDeA	Sm-PFOS	Total PFOS
Unweighted Sample Size	1474	1474	1474	1474	1474	1474	715	715
**Age (years)**								
12–13	2.85 (0.15)	1.62 (0.07)	1.36 (0.12)	0.71 (0.05)	0.23 (0.03)	0.16 (0.01)	0.93 (0.13)	3.18 (0.33)
14–15	3.26 (0.24)	1.52 (0.06)	1.91 (0.22)	0.68 (0.05)	0.18 (0.02)	0.15 (0.01)	0.81 (0.05)	3.14 (0.22)
16–17	3.06 (0.13)	1.49 (0.06)	1.39 (0.09)	0.63 (0.04)	0.18 (0.02)	0.17 (0.01)	0.88 (0.05)	3.28 (0.17)
18–20	3.83 (0.21)	1.75 (0.07)	1.85 (0.12)	0.66 (0.04)	0.18 (0.01)	0.19 (0.01)	1.05 (0.06)	3.70 (0.20)
*p* value	0.001	0.012	0.004	0.509	0.352	0.002	0.021	0.089
**Race and ethnicity**								
Mexican American	2.62 (0.15)	1.36 (0.06)	1.00 (0.06)	0.59 (0.04)	0.15 (0.02)	0.16 (0.01)	0.79 (0.05)	2.77 (0.20)
Other Hispanic	2.24 (0.17)	1.39 (0.06)	0.95 (0.11)	0.59 (0.05)	0.12 (0.01)	0.15 (0.01)	0.61 (0.04)	2.32 (0.15)
Non-Hispanic White	3.46 (0.16)	1.72 (0.06)	1.96 (0.16)	0.69 (0.04)	0.21 (0.01)	0.17 (0.01)	1.06 (0.06)	3.68 (0.18)
Non-Hispanic Black	3.66 (0.30)	1.44 (0.06)	1.76 (0.22)	0.63 (0.04)	0.18 (0.02)	0.16 (0.01)	0.75 (0.06)	3.11 (0.30)
Other/multiracial	4.11 (0.37)	1.86 (0.12)	1.39 (0.19)	0.78 (0.07)	0.21 (0.03)	0.25 (0.02)	0.89 (0.08)	3.78 (0.38)
*p* value	<0.001	<0.001	<0.001	0.096	<0.001	<0.001	<0.001	<0.001
**Sex**								
Male	3.73 (0.16)	1.74 (0.05)	1.97 (0.14)	0.71 (0.04)	0.20 (0.01)	0.17 (0.01)	1.05 (0.04)	3.84 (0.17)
Female	2.89 (0.12)	1.47 (0.05)	1.32 (0.08)	0.63 (0.04)	0.18 (0.01)	0.17 (0.01)	0.79 (0.06)	2.80 (0.15)
*p* value	<0.001	<0.001	<0.001	0.036	0.090	0.670	<0.001	<0.001
**BMI category (CDC criteria)**								
Underweight	3.04 (0.22)	1.59 (0.13)	1.32 (0.22)	0.58 (0.06)	0.19 (0.02)	0.18 (0.02)	0.93 (0.13)	3.63 (0.42)
Normal	3.62 (0.17)	1.69 (0.06)	1.87 (0.13)	0.69 (0.04)	0.19 (0.02)	0.18 (0.01)	1.00 (0.07)	3.66 (0.25)
Overweight	3.10 (0.14)	1.64 (0.05)	1.65 (0.17)	0.71 (0.06)	0.20 (0.02)	0.16 (0.01)	0.85 (0.05)	2.99 (0.15)
Obesity	2.89 (0.18)	1.43 (0.06)	1.31 (0.10)	0.60 (0.03)	0.17 (0.01)	0.15 (0.01)	0.82 (0.04)	2.90 (0.16)
Missing/unclassifiable	3.19 (0.40)	1.53 (0.23)	1.19 (0.23)	0.56 (0.06)	0.20 (0.03)	0.18 (0.02)	0.86 (0.16)	3.40 (0.40)
*p* value	0.009	0.005	0.001	0.121	0.327	0.024	0.175	0.093
**Household education**								
Less than high school	2.98 (0.16)	1.42 (0.06)	1.18 (0.07)	0.65 (0.05)	0.19 (0.02)	0.16 (0.01)	0.80 (0.06)	2.89 (0.23)
High school/some college	3.31 (0.15)	1.60 (0.06)	1.72 (0.11)	0.64 (0.03)	0.17 (0.01)	0.17 (0.01)	0.86 (0.05)	3.21 (0.14)
College or above	3.54 (0.17)	1.77 (0.05)	1.81 (0.17)	0.76 (0.06)	0.23 (0.02)	0.18 (0.01)	1.18 (0.09)	4.01 (0.31)
Missing/not reported	4.02 (0.44)	1.79 (0.16)	2.17 (0.41)	0.62 (0.05)	0.21 (0.05)	0.20 (0.03)	1.05 (0.11)	3.98 (0.57)
*p* value	0.021	<0.001	<0.001	0.072	0.054	0.096	<0.001	0.007
**Family income**								
<$20,000	3.39 (0.32)	1.63 (0.09)	1.77 (0.24)	0.64 (0.03)	0.19 (0.02)	0.17 (0.01)	0.86 (0.05)	3.13 (0.19)
$20,000–$44,999	3.14 (0.18)	1.49 (0.06)	1.48 (0.12)	0.62 (0.04)	0.19 (0.02)	0.17 (0.01)	0.86 (0.05)	3.13 (0.16)
$45,000–$74,999	3.00 (0.18)	1.54 (0.07)	1.38 (0.12)	0.67 (0.05)	0.17 (0.01)	0.16 (0.01)	0.86 (0.05)	3.18 (0.20)
≥$75,000	3.56 (0.18)	1.73 (0.06)	1.90 (0.21)	0.73 (0.05)	0.20 (0.01)	0.17 (0.01)	1.06 (0.07)	3.71 (0.22)
Missing/not classifiable	3.57 (0.42)	1.64 (0.13)	1.62 (0.30)	0.61 (0.06)	0.16 (0.02)	0.20 (0.02)	0.85 (0.16)	3.51 (0.64)
*p* value	0.099	0.035	0.074	0.150	0.403	0.818	0.019	0.036
**Vitamin C status**								
Deficient < 11.4 µmol/L	2.05 (0.14)	0.93 (0.13)	0.85 (0.11)	0.76 (0.20)	0.27 (0.04)	0.18 (0.02)	0.74 (0.11)	2.79 (0.24)
Non-deficient ≥ 11.4 µmol/L	2.42 (0.18)	1.28 (0.05)	1.33 (0.15)	0.48 (0.05)	0.20 (0.01)	0.18 (0.01)	0.90 (0.07)	3.32 (0.24)
*p* value	0.129	0.021	0.008	0.177	0.133	0.944	0.213	0.137
**Total cholesterol (mg/dL)**								
1st tertile	3.03 (0.17)	1.60 (0.07)	1.56 (0.10)	0.67 (0.05)	0.19 (0.01)	0.16 (0.01)	0.87 (0.05)	3.15 (0.20)
2nd tertile	3.53 (0.17)	1.62 (0.06)	1.80 (0.16)	0.63 (0.04)	0.20 (0.01)	0.18 (0.01)	1.00 (0.07)	3.57 (0.19)
3rd tertile	3.44 (0.21)	1.63 (0.05)	1.64 (0.13)	0.70 (0.04)	0.18 (0.01)	0.17 (0.01)	0.90 (0.04)	3.34 (0.20)
Missing	2.12 (0.09)	1.20 (0.00)	1.12 (0.09)	0.40 (0.11)	0.34 (0.07)	0.20 (0.00)	0.84 (0.04)	2.96 (0.04)
*p* value	0.069	0.918	0.208	0.219	0.364	0.672	0.105	0.114
**Cotinine (ng/mL)**								
<1	3.35 (0.13)	1.62 (0.04)	1.66 (0.09)	0.68 (0.04)	0.18 (0.01)	0.17 (0.01)	0.92 (0.04)	3.35 (0.15)
≥1 and <10	3.01 (0.23)	1.50 (0.08)	1.52 (0.25)	0.68 (0.05)	0.22 (0.04)	0.15 (0.01)	0.88 (0.10)	3.01 (0.28)
≥10	3.40 (0.23)	1.66 (0.08)	1.77 (0.18)	0.60 (0.03)	0.25 (0.03)	0.16 (0.01)	1.04 (0.09)	3.74 (0.30)
*p* value	0.348	0.143	0.750	0.168	0.051	0.315	0.349	0.255

Values are survey-weighted means (SE) in ng/mL. The six primary PFASs describe NHANES 2011–2018 (*n* = 1474); Sm-PFOS and total PFOS describe 2015–2018 (*n* = 715). Vitamin C summaries use the same measured 2017–2018 subset for all eight PFAS (*n* = 332). *p*-values compare observed levels within each characteristic; missing categories are excluded from these comparisons. Missing covariates are shown descriptively and were multiply imputed for adjusted regression. Italic *p* denotes a *p*-value.

**Table 2:** Survey-weighted associations of eight PFAS measures per one-unit increase in natural-log concentration with total bilirubin and albumin, overall and by vitamin C status.

Biomarker	PFAS	Overall Crude β (95% CI) *p*	Overall Adjusted β (95% CI) *p*; BH *q*	Vitamin C Deficient <11.4 µmol/L β (95% CI)	Vitamin C Non-deficient ≥11.4 µmol/L β (95% CI)	Interaction β (95% CI)	Interaction p; BH *q*
Total bilirubin (mg/dL)	PFOS	0.098 (0.068, 0.127) *p* < 0.001	0.028 (−0.001, 0.058) *p* = 0.061; *q* = 0.081	−0.728 (−1.862, 0.406)	−0.005 (−0.074, 0.064)	0.723 (−0.406, 1.852)	*p* = 0.190; *q* = 0.457
	PFOA	0.143 (0.107, 0.178) *p* < 0.001	0.057 (0.026, 0.089) *p* < 0.001; *q* = 0.002	−0.073 (−0.298, 0.151)	−0.001 (−0.069, 0.067)	0.073 (−0.161, 0.306)	*p* = 0.514; *q* = 0.686
	PFHxS	0.068 (0.048, 0.089) *p* < 0.001	0.026 (0.006, 0.047) *p* = 0.012; *q* = 0.024	−0.070 (−0.395, 0.255)	−0.013 (−0.054, 0.029)	0.057 (−0.248, 0.363)	*p* = 0.692; *q* = 0.755
	PFNA	0.072 (0.045, 0.099) *p* < 0.001	0.013 (−0.014, 0.040) *p* = 0.327; *q* = 0.392	0.034 (−0.203, 0.271)	0.022 (−0.045, 0.088)	−0.012 (−0.274, 0.250)	*p* = 0.921; *q* = 0.921
	Me-PFOSA-AcOH	0.002 (−0.017, 0.022) *p* = 0.810	−0.009 (−0.030, 0.013) *p* = 0.432; *q* = 0.472	−0.274 (−0.826, 0.279)	−0.003 (−0.074, 0.068)	0.271 (−0.255, 0.797)	*p* = 0.287; *q* = 0.492
	PFDeA	0.062 (0.027, 0.096) *p* < 0.001	0.047 (0.014, 0.081) *p* = 0.007; *q* = 0.017	−0.110 (−0.629, 0.408)	0.034 (−0.068, 0.136)	0.144 (−0.397, 0.684)	*p* = 0.576; *q* = 0.691
	Sm-PFOS	0.061 (0.023, 0.098) *p* = 0.002	0.003 (−0.036, 0.042) *p* = 0.869; *q* = 0.869	−0.336 (−0.777, 0.105)	−0.008 (−0.065, 0.050)	0.329 (−0.105, 0.762)	*p* = 0.125; *q* = 0.250
	Total PFOS	0.060 (0.017, 0.103) *p* = 0.007	0.014 (−0.031, 0.060) *p* = 0.530; *q* = 0.706	−0.749 (−1.600, 0.103)	−0.005 (−0.075, 0.064)	0.743 (−0.101, 1.587)	*p* = 0.080; *q* = 0.250
Albumin (g/L)	PFOS	0.794 (0.434, 1.154) *p* < 0.001	0.399 (0.076, 0.721) *p* = 0.016; *q* = 0.028	−1.689 (−7.222, 3.845)	0.315 (−0.227, 0.856)	2.003 (−3.664, 7.671)	*p* = 0.459; *q* = 0.686
	PFOA	1.641 (1.167, 2.115) *p* < 0.001	1.025 (0.630, 1.421) *p* < 0.001; *q* < 0.001	2.276 (1.441, 3.111)	0.544 (−0.169, 1.257)	−1.731 (−2.723, −0.740)	*p* = 0.002; *q* = 0.013
	PFHxS	0.623 (0.404, 0.842) *p* < 0.001	0.246 (0.035, 0.456) *p* = 0.023; *q* = 0.034	1.881 (−0.993, 4.755)	0.392 (0.044, 0.741)	−1.489 (−4.266, 1.288)	*p* = 0.268; *q* = 0.492
	PFNA	0.904 (0.598, 1.211) *p* < 0.001	0.417 (0.117, 0.716) *p* = 0.007; *q* = 0.017	1.479 (0.746, 2.212)	0.106 (−0.230, 0.442)	−1.373 (−2.070, −0.676)	*p* < 0.001; *q* = 0.011
	Me-PFOSA-AcOH	0.280 (0.002, 0.559) *p* = 0.048	0.081 (−0.147, 0.309) *p* = 0.479; *q* = 0.479	−2.404 (−5.196, 0.387)	0.086 (−0.351, 0.524)	2.491 (−0.411, 5.392)	*p* = 0.086; *q* = 0.259
	PFDeA	0.540 (0.168, 0.911) *p* = 0.005	0.655 (0.363, 0.947) *p* < 0.001; *q* < 0.001	4.631 (1.784, 7.478)	0.001 (−0.447, 0.449)	−4.630 (−7.400, −1.859)	*p* = 0.003; *q* = 0.013
	Sm-PFOS	1.520 (1.001, 2.039) *p* < 0.001	0.635 (0.207, 1.062) *p* = 0.005; *q* = 0.014	1.830 (−1.928, 5.587)	0.174 (−0.295, 0.643)	−1.656 (−5.408, 2.096)	*p* = 0.357; *q* = 0.476
	Total PFOS	1.286 (0.735, 1.836) *p* < 0.001	0.597 (0.177, 1.018) *p* = 0.007; *q* = 0.014	−0.361 (−6.640, 5.918)	0.300 (−0.226, 0.825)	0.661 (−5.702, 7.024)	*p* = 0.826; *q* = 0.826

Overall models used *n* = 1474 for the six primary PFASs and *n* = 715 (2015–2018) for Sm-PFOS and total PFOS. β (95% CI) denotes change in bilirubin (mg/dL) or albumin (g/L) per unit ln(PFAS). Adjusted models included age, sex, race/ethnicity, household-reference education, family income, cotinine, cholesterol, and survey cycle, with PFAS subsample weights, strata, and PSUs. Missing covariates were handled by 50 imputations. Benjamini–Hochberg (BH) correction was separate for 12 primary and four secondary adjusted continuous tests (*q* < 0.05). Vitamin C models used *n* = 332: <11.4 µmol/L and ≥11.4 µmol/L. All eight PFAS measures have slopes from pooled interaction models with common covariate coefficients and the same adjustment, except cycle. Interaction *q*-values use separate 12-test primary and four-test secondary families; group-specific slopes were not FDR-adjusted. The deficient group is small, and the comparisons are exploratory.

## Data Availability

The data are publicly available from the National Health and Nutrition Examination Survey (NHANES): https://www.cdc.gov/nchs/nhanes/ (accessed on 7 October 2026). Supporting results are presented in the manuscript and Supplementary Materials.

## Supplementary Materials

The following supporting information can be downloaded at: https://www.mdpi.com/article/10.3390/antiox15101310/s1, Table S1. Observed serum PFAS category ranges (ng/mL) and participant counts used in Figure 1, NHANES 2011–2018; Table S2. Unweighted participant characteristics by NHANES survey cycle, 2011–2018 (*n* = 1474); Table S3. Unweighted serum biomarker and PFAS concentrations by NHANES survey cycle, 2011–2018; Table S4. Unweighted Spearman correlations among eight serum PFAS exposure measures, NHANES 2011–2018; Table S5. Posterior inclusion probabilities for six serum PFAS in unweighted BKMR models of total bilirubin and albumin (*n* = 1474); Table S6. Sensitivity analysis of survey-weighted PFAS associations with total bilirubin and albumin using LOD/2 substitution for concentrations below the detection limit, NHANES 2011–2018; Figure S1. Participant selection for the final sample.
