# Supplementary material for: Potential Role of Vitamin C in the Associations of Serum Per- and Polyfluoroalkyl Substances with Bilirubin and Albumin in U.S. Adolescents and Young Adults: NHANES 2011–2018
Source: Antioxidants (Basel). 2026 Oct 12;15(10):1310. doi: 10.3390/antiox15101310 (PMC13700904; doi:10.3390/antiox15101310)
Supplement: Supplementary file 1 [file antioxidants-15-01310-s001.zip › antioxidants-4529230-supplementary.pdf]

Supplementary Materials

Potential Role of Vitamin C in the Associations of Serum Per- and Polyfluoroalkyl Substances with Bilirubin and Albumin in U.S. Adolescents and Young Adults: NHANES 2011–2018

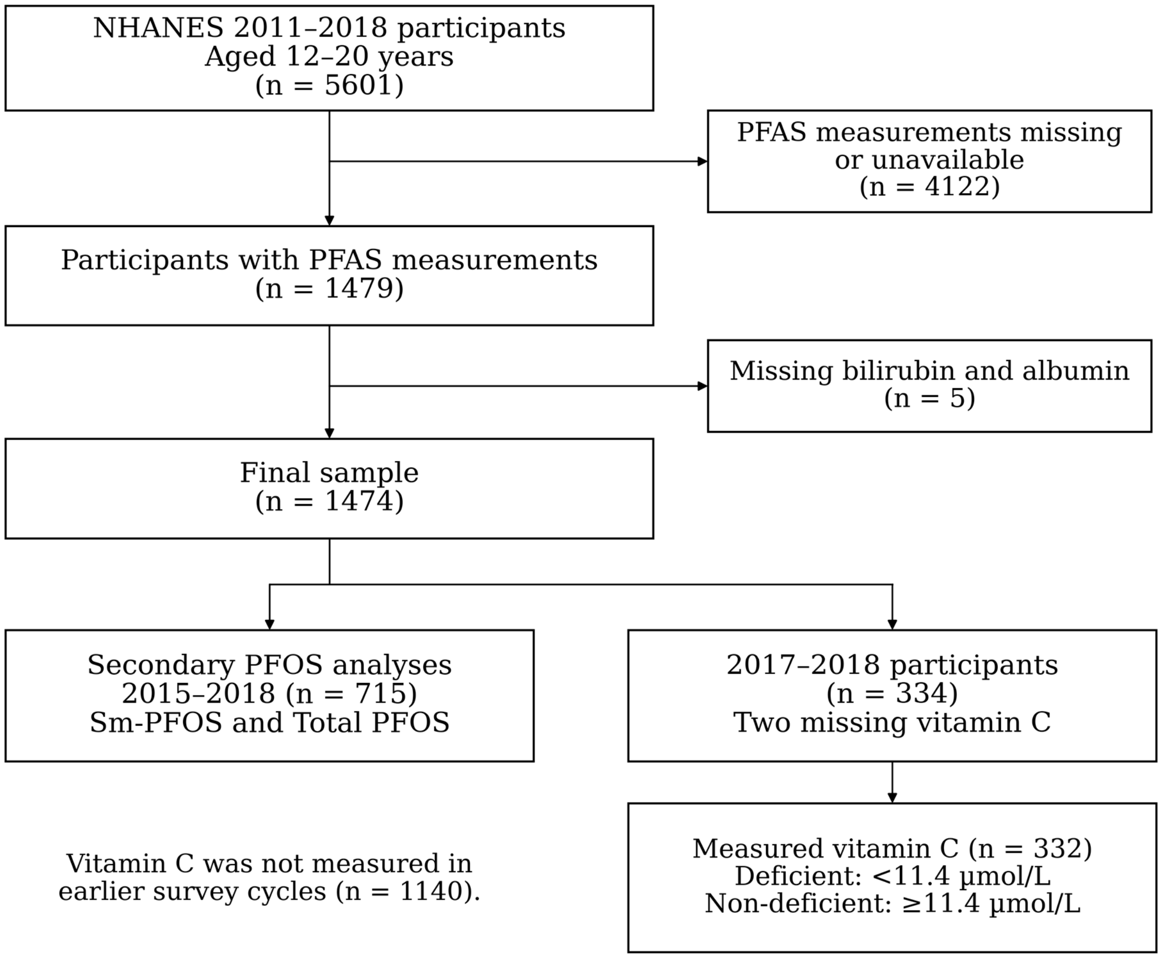

Figure S1. Participant selection for the final sample

Counts and percentages are unweighted. Among 5601 NHANES participants aged 12–20 years in 2011–2018, 1479 had complete PFAS measurements. Five participants missing both bilirubin and albumin were excluded, leaving a final sample of 1474. Of the 334 participants from 2017–2018 in the final sample, 332 had measured vitamin C.

Table S1. Observed serum PFAS category ranges (ng/mL) and participant counts used in Figure 1, NHANES 2011–2018

PFAS	Low range (*n*)	Medium range (*n*)	High range (*n*)
PFOS	0.07–1.8 (*n* = 510)	1.83–3.3 (*n* = 475)	3.32–43.9 (*n* = 489)
PFOA	0.07–1.08 (*n* = 491)	1.09–1.69 (*n* = 482)	1.7–14.1 (*n* = 501)
PFHxS	0.07–0.68 (*n* = 483)	0.7–1.29 (*n* = 495)	1.3–28.3 (*n* = 496)
PFNA	0.07–0.4 (*n* = 558)	0.41–0.68 (*n* = 394)	0.7–8 (*n* = 522)
Me-PFOSA-AcOH	0.06–0.07 (*n* = 738)	0.09–0.1 (*n* = 247)	0.11–3.29 (*n* = 489)
PFDeA	0.07–0.07 (*n* = 412)	0.1–0.19 (*n* = 483)	0.2–2.2 (*n* = 579)
Sm-PFOS	0.07–0.5 (*n* = 232)	0.6–0.9 (*n* = 267)	1–4.9 (*n* = 216)
Total PFOS	0.14–2 (*n* = 240)	2.1–3.2 (*n* = 237)	3.3–46 (*n* = 238)

Concentrations are ng/mL; *n* is the unweighted category count in the final sample. The six primary PFAS use NHANES 2011–2018 (*n* = 1474); Sm-PFOS and Total PFOS use 2015–2018 (*n* = 715). All eight exposure measures are included in the conventional analyses in Table 2 and Figures 1–2. Categories were defined using mean ranks for ties; unequal category sizes reflect ties. Me-PFOSA-AcOH denotes 2-(N-methyl-perfluorooctane sulfonamido) acetic acid.

Table S2. Unweighted participant characteristics by NHANES survey cycle, 2011–2018 (*n* = 1474)

Characteristic	2011–2012	2013–2014	2015–2016	2017–2018	Total
Participants, *n*	375	384	381	334	1474
Age, mean (SD), years	15.89 (2.54)	16.02 (2.48)	15.69 (2.45)	16.08 (2.41)	15.91 (2.47)
Race and ethnicity					
Mexican American	65 (17.3)	90 (23.4)	88 (23.1)	74 (22.2)	317 (21.5)
Other Hispanic	32 (8.5)	39 (10.2)	50 (13.1)	25 (7.5)	146 (9.9)
Non-Hispanic White	92 (24.5)	114 (29.7)	107 (28.1)	102 (30.5)	415 (28.2)
Non-Hispanic Black	115 (30.7)	89 (23.2)	75 (19.7)	68 (20.4)	347 (23.5)
Other/multiracial	71 (18.9)	52 (13.5)	61 (16.0)	65 (19.5)	249 (16.9)
Sex					
Male	191 (50.9)	201 (52.3)	209 (54.9)	176 (52.7)	777 (52.7)
Female	184 (49.1)	183 (47.7)	172 (45.1)	158 (47.3)	697 (47.3)
Household education					
Less than high school	95 (25.3)	93 (24.2)	94 (24.7)	67 (20.1)	349 (23.7)
High school/some college	199 (53.1)	211 (54.9)	199 (52.2)	195 (58.4)	804 (54.5)
College or above	63 (16.8)	75 (19.5)	68 (17.8)	54 (16.2)	260 (17.6)
Missing/not reported	18 (4.8)	5 (1.3)	20 (5.2)	18 (5.4)	61 (4.1)
Family income					
<$20,000	96 (25.6)	80 (20.8)	78 (20.5)	60 (18.0)	314 (21.3)
$20,000–$44,999	108 (28.8)	124 (32.3)	105 (27.6)	101 (30.2)	438 (29.7)
$45,000–$74,999	53 (14.1)	60 (15.6)	78 (20.5)	60 (18.0)	251 (17.0)
≥$75,000	89 (23.7)	96 (25.0)	91 (23.9)	78 (23.4)	354 (24.0)
Missing/not reported	29 (7.7)	24 (6.2)	29 (7.6)	35 (10.5)	117 (7.9)
BMI category					
Underweight	9 (2.4)	13 (3.5)	13 (3.5)	15 (4.6)	50 (3.5)
Normal	207 (56.1)	208 (55.3)	197 (52.7)	171 (52.3)	783 (54.1)
Overweight	63 (17.1)	70 (18.6)	73 (19.5)	46 (14.1)	252 (17.4)
Obesity	90 (24.4)	85 (22.6)	91 (24.3)	95 (29.1)	361 (25.0)
Missing/unclassifiable	6 (1.6)	8 (2.1)	7 (1.8)	7 (2.1)	28 (1.9)

Values are observed *n* (column %) unless indicated; total *n* = 1474. Missing/not reported values are shown before imputation. BMI categories use CDC BMI-for-age percentiles at ages 12–19 (<5th, 5th to <85th, 85th to <95th, and ≥95th percentiles) and adult BMI cut points at age 20 (<18.5, 18.5 to <25, 25 to <30, and ≥30 kg/m^2). BMI-category percentages use classifiable participants; the missing row uses the full cohort denominator. BMI was unavailable for 16 participants; 12 additional participants aged 19 lacked age in months, giving 28 unclassifiable categories.

Table S3. Unweighted serum biomarker and PFAS concentrations by NHANES survey cycle, 2011–2018

Parameter	2011–2012	2013–2014	2015–2016	2017–2018
Total bilirubin (mg/dL)	0.70 (0.31)<br>*n* = 375	0.66 (0.32)<br>*n* = 384	0.53 (0.29)<br>*n* = 381	0.46 (0.32)<br>*n* = 334
Albumin (g/L)	44.42 (3.07)<br>*n* = 375	44.67 (3.09)<br>*n* = 384	45.22 (3.16)<br>*n* = 381	42.75 (3.22)<br>*n* = 334
PFOS (ng/mL)	5.10 (3.71)<br>*n* = 375	3.25 (3.08)<br>*n* = 384	2.42 (1.78)<br>*n* = 381	2.37 (2.98)<br>*n* = 334
PFOA (ng/mL)	1.93 (0.92)<br>*n* = 375	1.66 (0.89)<br>*n* = 384	1.37 (1.18)<br>*n* = 381	1.22 (0.97)<br>*n* = 334
PFHxS (ng/mL)	1.77 (2.58)<br>*n* = 375	1.81 (2.55)<br>*n* = 384	1.07 (1.06)<br>*n* = 381	1.20 (1.83)<br>*n* = 334
PFNA (ng/mL)	0.93 (0.57)<br>*n* = 375	0.74 (0.67)<br>*n* = 384	0.57 (0.35)<br>*n* = 381	0.43 (0.37)<br>*n* = 334
Me-PFOSA-AcOH (ng/mL)	0.19 (0.28)<br>*n* = 375	0.17 (0.23)<br>*n* = 384	0.17 (0.26)<br>*n* = 381	0.16 (0.19)<br>*n* = 334
PFDeA (ng/mL)	0.18 (0.12)<br>*n* = 375	0.18 (0.18)<br>*n* = 384	0.15 (0.18)<br>*n* = 381	0.18 (0.17)<br>*n* = 334
Sm-PFOS (ng/mL)	—	—	0.90 (0.52)<br>*n* = 381	0.79 (0.57)<br>*n* = 334
Total PFOS (ng/mL)	—	—	3.32 (2.13)<br>*n* = 381	3.16 (3.34)<br>*n* = 334
Total cholesterol (mg/dL)	155.91 (29.30)<br>*n* = 375	156.32 (29.61)<br>*n* = 384	156.66 (29.74)<br>*n* = 381	157.52 (28.81)<br>*n* = 332
Cotinine, *n* (%)				
<1 ng/mL	297 (79.2)	303 (78.9)	306 (80.3)	276 (82.6)
≥1 and <10 ng/mL	30 (8.0)	39 (10.2)	36 (9.4)	26 (7.8)
≥10 ng/mL	48 (12.8)	42 (10.9)	39 (10.2)	32 (9.6)

Continuous values are observed mean (SD), followed by available *n*. Cotinine values are *n* (column %). Sm-PFOS and Total PFOS summaries were restricted to 2015–2018; separately released 2013–2014 surplus-isomer variables were not incorporated in the secondary exposure definitions. PFOS and PFOA represent total concentrations in 2011–2012 and linear-isomer concentrations in later cycles. Total PFOS equals linear PFOS plus Sm-PFOS. An em dash indicates a cycle not included in the secondary summaries.

Table S4. Unweighted Spearman correlations among eight serum PFAS exposure measures, NHANES 2011–2018

PFAS	PFOS	PFOA	PFHxS	PFNA	Me-PFOSA-AcOH	PFDeA	Sm-PFOS	Total PFOS
PFOS	1.000	0.620 (<0.001)	0.555 (<0.001)	0.585 (<0.001)	0.192 (<0.001)	0.535 (<0.001)	0.765 (<0.001)	0.985 (<0.001)
PFOA	0.620 (<0.001)	1.000	0.565 (<0.001)	0.643 (<0.001)	0.098 (<0.001)	0.455 (<0.001)	0.652 (<0.001)	0.575 (<0.001)
PFHxS	0.555 (<0.001)	0.565 (<0.001)	1.000	0.341 (<0.001)	0.151 (<0.001)	0.152 (<0.001)	0.686 (<0.001)	0.617 (<0.001)
PFNA	0.585 (<0.001)	0.643 (<0.001)	0.341 (<0.001)	1.000	0.027 (0.307)	0.511 (<0.001)	0.524 (<0.001)	0.534 (<0.001)
Me-PFOSA-AcOH	0.192 (<0.001)	0.098 (<0.001)	0.151 (<0.001)	0.027 (0.307)	1.000	0.098 (<0.001)	0.345 (<0.001)	0.339 (<0.001)
PFDeA	0.535 (<0.001)	0.455 (<0.001)	0.152 (<0.001)	0.511 (<0.001)	0.098 (<0.001)	1.000	0.236 (<0.001)	0.417 (<0.001)
Sm-PFOS	0.765 (<0.001)	0.652 (<0.001)	0.686 (<0.001)	0.524 (<0.001)	0.345 (<0.001)	0.236 (<0.001)	1.000	0.857 (<0.001)
Total PFOS	0.985 (<0.001)	0.575 (<0.001)	0.617 (<0.001)	0.534 (<0.001)	0.339 (<0.001)	0.417 (<0.001)	0.857 (<0.001)	1.000

Cells show unweighted Spearman correlation coefficients, with two-sided *p*-values in parentheses; *n* = 1474 for pairs of primary PFAS and *n* = 715 for any pair involving Sm-PFOS or Total PFOS (2015–2018). P-values are unadjusted and descriptive.

Table S5. Posterior inclusion probabilities for six serum PFAS in unweighted BKMR models of total bilirubin and albumin (*n* = 1474)

PFAS	Total bilirubin	Albumin
PFOS	0.564	0.164
PFOA	0.758	1.000
PFHxS	0.550	0.052
PFNA	0.534	0.026
Me-PFOSA-AcOH	0.428	0.038
PFDeA	0.922	0.078

Unweighted BKMR used *n* = 1474 per outcome and six standardized log-PFAS, with standardized outcomes. Values are from the reporting fit in the first completed imputation and match Figure 3. A second completed imputation was fitted separately as a sensitivity analysis. These are not estimates pooled across 50 imputations. PIPs quantify relative inclusion conditional on the other mixture components; they are not effect sizes or causal contributions. Bilirubin kernel-scale effective sample sizes were only 14.3 and 7.4 out of 500 retained draws in the reporting and sensitivity fits, respectively. Bilirubin PIPs also varied across completed datasets, limiting confidence in their ranking and in the mixture summaries.

Table S6. Sensitivity analysis of survey-weighted PFAS associations with total bilirubin and albumin using LOD/2 substitution for concentrations below the detection limit, NHANES 2011–2018

Outcome	PFAS	*n*	β (95% CI)	*p*	BH *q*
Total bilirubin	PFOS	1474	0.028 (−0.001, 0.057)	0.062	0.082
Total bilirubin	PFOA	1474	0.057 (0.025, 0.088)	<0.001	0.002
Total bilirubin	PFHxS	1474	0.026 (0.006, 0.046)	0.013	0.026
Total bilirubin	PFNA	1474	0.013 (−0.014, 0.040)	0.339	0.407
Total bilirubin	Me-PFOSA-AcOH	1474	−0.007 (−0.026, 0.013)	0.491	0.515
Total bilirubin	PFDeA	1474	0.041 (0.015, 0.068)	0.003	0.009
Total bilirubin	Sm-PFOS	715	0.003 (−0.035, 0.041)	0.878	0.878
Total bilirubin	Total PFOS	715	0.014 (−0.031, 0.058)	0.534	0.712
Albumin	PFOS	1474	0.392 (0.069, 0.715)	0.018	0.031
Albumin	PFOA	1474	1.008 (0.603, 1.413)	<0.001	<0.001
Albumin	PFHxS	1474	0.241 (0.033, 0.449)	0.024	0.036
Albumin	PFNA	1474	0.412 (0.124, 0.700)	0.006	0.014
Albumin	Me-PFOSA-AcOH	1474	0.064 (−0.131, 0.259)	0.515	0.515
Albumin	PFDeA	1474	0.595 (0.346, 0.844)	<0.001	<0.001
Albumin	Sm-PFOS	715	0.617 (0.189, 1.045)	0.006	0.020
Albumin	Total PFOS	715	0.579 (0.151, 1.007)	0.010	0.020

β (95% CI) is the change in total bilirubin (mg/dL) or albumin (g/L) per one-unit increase in ln(PFAS). Values below the limit of detection (LOD) were replaced by LOD/2. Models retained the primary covariates, survey design, and 50 imputations. BH *q* is the Benjamini–Hochberg adjusted *p*-value, calculated separately for 12 primary and four secondary tests; $q < 0.05$ indicates significance. Sm-PFOS and Total PFOS analyses used NHANES 2015–2018 ($n = 715$).
